# Effects of Anesthetics on Barrier Tissue Function

**DOI:** 10.1155/2019/5920620

**Published:** 2019-10-21

**Authors:** Fujing Wang, Yanhui Li, Changlei Cui, Zhaoping Xue, Haichun Ma

**Affiliations:** ^1^Department of Anesthesia, The First Hospital of Jilin University, No. 71 Xinmin Street, Changchun, Jilin Province 130021, China; ^2^Postanesthesia Care Unit, The First Hospital of Jilin University, No. 71 Xinmin Street, Changchun, Jilin Province 130021, China

## Abstract

Anesthetics have long been proven to have additional effects other than anesthesia on different organs and tissues of the human body. Barrier tissues play critical roles in human health and diseases, yet the impacts of anesthetics on barrier tissues are still not clear. This review article is aimed at summarizing different effects of anesthetics on the skin, the respiratory, and intestinal membranes from two aspects: inflammation/immunity and ischemia-reperfusion. Among volatile, intravenous, and local anesthetics, volatile anesthetics are less influential on barrier ischemia-perfusion function. Although direct comparisons between volatile and the other two types of anesthetics are still lacking, volatile anesthetics appear to have stronger anti-inflammatory effects on different barrier tissues through various mechanisms. These results suggested that when treating patients with barrier tissue complications, volatile anesthetics can provide better therapeutic outcomes.

## 1. Introduction

Barrier tissues, as the first line of the protection system in living organisms, are constantly exposed to harmful components. The respiratory and intestinal mucous membranes and the skin defend the body against various biological, chemical, and physical insults. This protection function appears to be more vital with critically ill patients. Therefore, clarifying the effects of different anesthetics on barrier tissues during the induction and maintenance of anesthesia becomes critical when surgical operations are necessary. This article is aimed at summarizing and classifying the effects on barrier tissues of agents commonly used during anesthesia. It will help us to choose appropriate anesthetics depending on the complications of the patient without doing unnecessary damage to the barrier tissues.

## 2. Volatile Anesthetics

Volatile anesthetics refer to agents that come into effect through inhalation, including nitrous oxide and a series of fluorinated liquids (such as sevoflurane, desflurane, isoflurane), the latter needs a specific vaporizer to transform the liquids into gases, and further lead to unconsciousness and muscle relaxant. Across all types of anesthetics, volatile anesthetics seem to be more effective at protecting both myocardial and respiratory cells [1]. According to Gargiulo et al., isoflurane has only a minor influence on the murine hemodynamic status [2], indicating that use of volatile anesthetics does not obviously reduce the blood flow in tissues. This ensures the tissues away from ischemia-reperfusion damage, which may lead to a cascade reaction including microthrombus, histohypoxia, and finally, cell damage or even cell death. Moreover, by modulating pulmonary epithelial cell secretion [3], volatile anesthetics help decrease the production and expression of inflammatory mediators including cytokine-induced neutrophil chemoattractant- (CINC-) 1 and monocyte chemoattractant protein- (MCP-) 1. Noticeably, by decreasing the expression of intercellular adhesion molecule- (ICAM-) 1 protein, which is an important mediator within the inflammatory cascade, volatile anesthetics help to avoid the adhesion of neutrophils by 71% and reduce the death rate of alveolar epithelial cells up to 26% [4]. Although the process is not clarified, it has been established that volatile anesthetics such as sevoflurane and isoflurane can reduce the neutrophil accumulation on alveolar epithelial cells and assist the attenuation of endotoxin-induced injury mediated by multiple cytokines and chemokines. Therefore, these agents can be a supporting therapy for patients with respiratory disease as both preconditioning and postconditioning.

As widely shown, volatile anesthetics are ideal for pediatric patients, due to its painless induction and fast metabolism. When using volatile anesthetics on infants, especially those with respiratory distress syndrome, special care is required because their pulmonary surfactant is not fully developed or already damaged. Paugam-Burtz et al.'s research has found that in mechanically ventilated in vivo rat models, volatile anesthetics may reduce the synthetize of pulmonary surfactant by affecting the content of lung SP-C mRNA [5]. This indicates that when anesthetizing infants with respiratory disease, intravenous anesthesia or intravenous inhaled balanced anesthesia may have less influence on respiratory function than inhaled anesthesia alone. In short, additional consideration is necessary when dealing with pediatric patients in regard to respiratory function.

Nitrous oxide, which was widely used as an anesthetic in humans decades ago, is well accepted for its short-acting analgesic properties. Studies have shown that through the depression of receptor-dependent generation of H_2_O_2_, nitrous oxide also has anti-inflammatory effects on cells by attenuating the normal functions of neutrophil and interfering the leukocyte adhesion-activation cascade [6, 7]. Regardless, when combining these components together (such as sevoflurane and nitrous oxide), the anti-inflammatory effects of sevoflurane and nitrous oxide are both eliminated, and the combination even induces the inflammatory response or suppresses the normal anti-inflammatory response [8].

Few researches have investigated the protective influence of volatile anesthetic agents on intestinal membrane. According to Liu et al., clinical relevant concentrations of sevoflurane have the ability to protect the intestinal mucous by attenuating the damage derived from intestinal ischemia-reperfusion injury [9]. Although the results on concentrations of volatile anesthetics may vary slightly due to different conditioning time points, we do know that ischemia on the intestinal mucous may lead to severe damage such as inflammation, dramatic hypotension, enterobrosis, or more seriously, intestinal perforation. Therefore, the potential to reverse the damage caused by ischemia-reperfusion in the alimentary canal endorses volatile anesthetics as an important therapy for patients suffering from digestive disorders.

## 3. Intravenous Anesthetics

Some anesthetics can be used intravenously, entering the blood circulation system to produce unconsciousness, sedation, analgesia, and muscle relaxant. Compared to volatile anesthetics, intravenous anesthetics lead to an obvious reduction in blood pressure postinjection, especially during the induction period requiring injection of more than two kinds of agents simultaneously. This is a common phenomenon among intravenous anesthetic agents, although they differ in the degree of hypotension they may cause. This is mainly due to the vasodilation effect, which increases the peripheral vascular volume. If the decrease in blood pressure is not managed or is excessive in duration, it can lower tissue oxygenation and culminate in ischemia. As performed by Abramovic et al., the pressure of oxygen (pO_2_) in the skin is much lower when using ketamine and xylazine to provide intravenous general anesthesia in vivo in rats, compared to the group using isoflurane for inhaled general anesthesia [10]. Noticeably, when hypotension develops during intravenous general anesthesia, ischemia occurs not only in the skin but also in all body tissues. This may be avoided by monitoring or observing the patient and using vasoactive agents if necessary. In addition, caution should be used when administering intravenous general anesthesia agents to patients with skin ischemic necrosis or other dermatosis.

Burn patients, unlike other dermatosis patients, usually have multiple organ dysfunctions as well as skin damage and inflammation simultaneously. Even though this group requires repeated surgeries under general anesthesia or local anesthesia, and the agents may have diverse effects on skin function, these reactions appear to be minor compared to therapies used for other treatment purposes. Similarly, most selective surgery inpatient or day surgery patients have adequate gastrointestinal preparation before the operation, which inhibits most inflammatory and immune events and thus prevents the effects of anesthetic agents as long as the gastrointestinal tract has sufficient blood supply. In the event the patient develops intestinal complications, in most instances, the complication itself induces inflammatory or immune responses greater than those caused by anesthetics. As these anesthetics tend to have minor side effects, there are very few papers on this subject. We deduced from the inflammatory and immune protective characters of anesthetics that they may have positive impacts on intestinal mucous and skin, but this hypothesis requires additional support from further research.

The alveolar type II pneumocyte cell synthesizes and restores the pulmonary surfactant, proliferates, and differentiates into type I pneumocyte cells [11], and is thereby the most significant cell type in the recovery from respiratory diseases such as acute lung injury. Agents that might weaken the function of the alveolar type II pneumocyte cell may, in effect, obstruct the normal function of respiratory membrane. In the experiment performed by Nishina et al., they tested different anesthesia agents' impacts on keratinocyte growth factors and hepatocyte growth factors; both have been shown to be the most potent mitogen for type II epithelial cells [11]. Those medications included midazolam, ketamine, thiopental, propofol, and lidocaine. As a result, they found that none of these agents in clinical relevant concentration had any influence on the proliferation of type II epithelial cells [12]. This suggests that most commonly used intravascular anesthesia agents do not impair the normal function of lung epithelial cells.

On the contrary, intravenous anesthetics showed a striking attenuation of the inflammatory response to protect the respiratory membrane. The disturbance of invasive surgical procedures like esophagectomy may have a drastically different result compared to minor operations such as lung resection, since big trauma incurs a more powerful inflammatory response at the airway and thus makes the moderating effect of anesthetic agents more apparent. According to the research of Wakabayashi et al., the inflammatory changes of the respiratory epithelial lining fluid (ELF) are more sensitive than those in the sera in esophagectomy. Within the ELF, the levels of inflammatory cytokines and chemokine including tumor necrosis factor- (TNF-) *α*, interleukin- (IL-) 1*β*, IL-6, IL-8, IL-10, and IL-12p70 were measured. TNF-*α*, IL-1*β*, and IL-6 serve as proinflammatory molecules while IL-8 serves as chemoattractant, and the IL-10 inhibits the production of proinflammatory cytokines and the normal antigen-presenting function. During the procedure, propofol showed a more potent suppression of the surgical stress-induced inflammatory perturbation in the ELF of the airway than sevoflurane, as the level of IL-6 and IL-8 in the propofol group was significantly lower than sevoflurane group, and the level of IL-10 on the contrary experienced an increase [13]. This may be the result of the esophagectomy, which is one of the most invasive operations in gastrointestinal surgeries. In another observation [14], elective lung resection was performed instead of esophagectomy when the anti-inflammatory effects of sevoflurane and propofol were observed. The result is quite different that sevoflurane showed a more pronounced anti-inflammatory effect and significantly suppressed the inflammatory response than propofol did. Thus, we can deduct that the inflammatory response caused by invasive surgeries can hardly be eliminated by anesthetics, especially when one-lung ventilation (OLV) is needed. The intrathoracic pressure changes (in both thoracoscope and open-chest procedure) and constricts the ventilated lung volume, in turn reducing the absorption and metabolism of volatile agents. Conversely, intravascular agents are not negatively impacted by the decreased lung volume and maintain a stable concentration.

However, though propofol showed a significant protective effect on normal cells, it is widely accepted that the concentration of propofol under any clinical circumstance should be within 5 mg/kg/h. The overdose and prolonged use of propofol may also lead to the cell death of microvascular and arterial endothelial cells, due to the activation of cathepsin D and glycogen synthase kinase- (GSK-) 3 [15], thereby impairing the normal blood supply of epithelial cell, resulting in the dysfunction of respiratory epithelium.

## 4. Local Anesthetic

Compared to intravenous and inhaled anesthetics, local anesthetics rarely enter into the blood flow and instead affect local tissues. They block the transmission of nerve impulses without causing changes in status of consciousness. Local anesthesia includes five main types: topical anesthesia, local infiltration anesthesia, nerve and plexus block, regional block, and intravenous local anesthesia; the first two kinds may impact the barrier tissue.

In the experiment performed by Ji et al., compared to topical anesthesia, subcutaneous infiltration anesthesia showed a wider range of tissue effects when used on the skin. It stimulates the tissue to generate new, thicker collagen fiber from the necrosis during the treatment of the plasma skin regeneration system, while topical anesthesia will reduce both skin necrosis and the collage fiber regeneration [16]. One explanation for this might be that when injecting subcutaneously, the agent slightly lifts the tissue, loosens the tissue structure, and further provokes the fibroblasts to generate more collagen fiber.

Although it has been established that the application of local anesthetics intravascularly or via surface infiltration will mitigate the airway response caused by tracheal intubation, it is unclear whether local anesthetics such as ropivacaine and lidocaine can trigger an anti-inflammatory mechanism in the pulmonary endothelial barrier. In trials run by Piegeler et al., they found that in rats, ropivacaine and lidocaine attenuate TNF-*α*-induced Src activation and endothelial nitric oxide synthase phosphorylation, equivalent to the blockage of the inflammatory TNF-*α* signal pathway [17]. This finding indicates that these two local anesthetics can prevent the pulmonary endothelial cells from an inflammatory response and further maintain the permeability of the pulmonary microvasculature. The normal function and blood supply of the pulmonary alveolar is therefore permitted. This experiment indicates that the surface infiltration of local anesthesia may improve both the recovery from acute inflammation and the stabilized stimulation caused by a tracheal tube.

## 5. Conclusion

**Figure 1:**
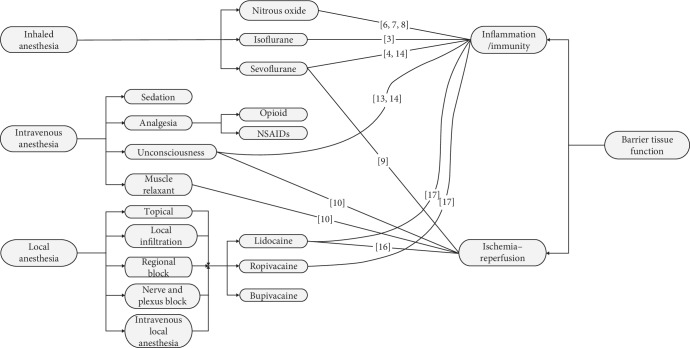
Major division and representative drug—function diagram. The main methods of anesthesia, the major classification of each, and the representative agents mentioned in the references were listed. The functions on barrier tissue of agents were classified, and the numbers refer to the references.

Figure 1 shows the major classification of anesthesia and the representative agents; their functions were classified with the reference number marked on the lines. To sum up, direct comparison of inhaled and intravenous anesthetics is not well studied as these two types of agents are usually used in different clinical settings except for invasive surgical procedures, although they are both proved to have anti-inflammatory effect in some degrees. Furthermore, researchers tend to indicate the development of inflammatory and immune response through diverse pathways, but we can draw a conclusion that inhaled anesthetics have more extensive anti-inflammatory response than the other two types, thus should be considered as a better option when less inflammation is desired in barrier tissues.

A different surgery type requires for different methods of anesthesia, and the real complications of patients should be the basis for the selection of anesthetic agents. Patients with barrier disorder are commonly encountered during clinical treatments and require greater observation of side effects and potential risks from various anesthetic agents. Appropriate selection and utilization of anesthetics and the combination of different anesthetic methods may help address and prevent these risks and further affect patient satisfaction and safety, shorten the hospital stay, and improve patient experience.
